# Banhasasim-Tang Attenuates Lipopolysaccharide-Induced Cognitive Impairment by Suppressing Neuroinflammation in Mice

**DOI:** 10.3390/nu12072019

**Published:** 2020-07-07

**Authors:** You-Chang Oh, Yun Hee Jeong, Malk Eun Pak, Younghoon Go

**Affiliations:** Korean Medicine (KM)-Application Center, Korea Institute of Oriental Medicine, Daegu 41062, Korea; ulivuli@kiom.re.kr (Y.-C.O.); runxi0333@kiom.re.kr (Y.H.J.); clear46@kiom.re.kr (M.E.P.)

**Keywords:** cognitive impairment, neuroinflammation, neuronal protection, Morris water maze, MAPK, banhasasim-tang

## Abstract

Banhasasim-tang (BHS) is an herbal medicine that has been widely used in East Asia to treat various symptoms associated with upper abdomen swelling. BHS has not been studied previously for neuroinflammation or cognitive disorder. Here, we use a lipopolysaccharide (LPS) model to investigate the effects and mechanisms of BHS in neuroinflammation and cognitive impairment of mice. We used a mouse model of LPS-induced cognitive impairment and neuroinflammation and examined whether administration of BHS prevents these deficits via Morris water maze test, passive avoidance test, histopathological analysis, Western blotting, and real-time reverse transcription polymerase chain reaction (RT-qPCR). We found via behavioral tests that BHS treatment effectively prevented LPS-induced memory loss and neuronal damage in mice. Histopathological analysis of mouse brains revealed that BHS inhibited LPS-induced expression of microglial and astrocyte activation markers. Furthermore, BHS inhibits the production of markers related to neurodegeneration, amyloidogenesis, and inflammation, and mRNA expression of inflammatory mediators in mouse brain tissue. Additionally, BHS pretreatment effectively inhibited generation of inflammatory factors and pathways in BV2 microglial cells stimulated by LPS. These observations indicate that BHS is effective in preventing cognitive impairment caused by neuroinflammation and has strong potential as a candidate treatment for neuronal inflammatory diseases.

## 1. Introduction

Neuroinflammation is a reported part of the neuropathogenesis of cognitive impairment [1]. It also is an important factor contributing to neurodegenerative diseases such as Alzheimer’s disease [2]. Microglia, resident macrophages in the brain, play an important role in the occurrence and development of neuroinflammation [3]. Under neuroinflammatory conditions, microglia are extensively activated, promoting the production of inflammatory mediators that further lead to astrocyte activation and neuronal damage. Meanwhile, the intra- and intercellular accumulation of abnormal aggregated proteins such as amyloid-β (Aβ) in injured neuronal tissue causes continuous neuroinflammation, as such proteins are capable of enhancing the activation of microglia and astrocytes [4].

Lipopolysaccharide (LPS), an immunostimulatory component of Gram-negative bacteria, was first identified as a ligand of toll-like receptor 4 (TLR-4) [5]. TLR-4 is primarily expressed on macrophages and microglia, which, once activated, produce various inflammatory mediators [6,7]. These substances are key mediators of the neuroinflammatory process in the central nervous system (CNS). Therefore, the injection of LPS into animals induces cognitive impairment and behavioral disorder including anorexia and decreased locomotion [8]. Because these symptoms are similar to those of human neurodegenerative diseases, the LPS model is frequently used to study neuroinflammation. Besides, LPS is widely used in various in vitro and in vivo tests in inflammation-related studies [9,10]. Additionally, indomethacin (Indo) is a representative of the nonsteroidal anti-inflammatory drug (NSAID) family that is widely used as an anti-inflammatory agent and is also used as a positive control in the study of the efficacy of systemic inflammation inhibition [11].

BHS is a traditional herbal decoction used in East Asian countries such as Korea, China, and Japan. It is listed *Dynamics of Shang Han Lun*, an ancient medical text. BHS is currently prescribed for the treatment of upper abdomen swelling and the resulting hiccups, nausea, vomiting, and diarrhea. A recent study demonstrated that BHS shows an anti-obesity effect in high fat diet induced obese mice and 3T3-L1 adipocytes [12]. Another previous study revealed that BHS has an anti-apoptotic effect on chronic acid reflux esophagitis [13]. However, thus far, no studies have explored the effects of BHS on cognitive impairment through neuroinflammation.

Therefore, in this study, we investigated the effects of a hot-water extract of BHS decoction on cognitive impairment caused by LPS injection in ICR mice. We examined the learning ability of LPS-injected ICR mice through behavioral tests and analyzed the pathological changes in mouse brain tissues and the expression of associated inflammatory mediators with or without BHS treatment. Indo, an anti-inflammatory agent, was used as a positive control for in vivo study. We also determined the inhibitory effects of BHS on the inflammatory response in LPS-stimulated brain microglia BV2 cells.

## 2. Materials and Methods

### 2.1. Materials and Reagents

BHS powder (Batch No. #18562) was obtained from Hanpoong Pharm. Co Ltd. (Wanju, Korea). The herbal ingredients and composition ratio of BHS decoction are indicated in Table 1. ICR mice (8 weeks old) were purchased from Samtako BioKorea (Osan, Korea). LPS (*E. coli* O55:B5), Indo, and dexamethasone (Dex) were acquired from Sigma-Aldrich (St. Louis, MO, USA). Dulbecco’s modified Eagle’s medium (DMEM), fetal bovine serum (FBS), antibiotics, bovine serum albumin (BSA), and enzyme-linked immunosorbent assay (ELISA) antibody kits were purchased from Hyclone (Logan, UT, USA) and eBioscience (#88-7324-88 and #88-7064-88, San Diego, CA, USA). Consumables used for cell culture, including dishes, well plates, and tubes, were acquired from Sarstedt (Nümbrecht, Germany). Cell counting kits (CCK) were obtained from Dojindo Molecular Technologies (Kumamoto, Japan). Various primary and secondary antibodies were acquired from Cell Signaling Technology (Danvers, MA, USA). A polyvinylidene fluoride (PVDF) membrane and enhanced chemiluminescence (ECL) solution were purchased from Millipore (Bedford, MA, USA). RNA extraction kits were obtained from iNtRON Biotechnology (Daejeon, Korea). DNA synthesis kits, oligonucleotide primers for RT-qPCR, and AccuPower 2X Greenstar qPCR Master Mix (ROX) were acquired from Bioneer (Daejeon, Korea). REAL EnVision detection systems were purchased from WAKO (K500711-2, Neuss, Germany).

### 2.2. Ethical Approval

All experimental procedures conducted in this study were performed in accordance with the guidelines for the Animal Care and Use Committee of Korea Institute of Oriental Medicine (Reference number #D-18-014). All efforts were made to minimize animal suffering and to reduce the number of animals used. Mice were housed four per cage with automatic temperature control, relative humidity, and a 12-h light–dark cycle. A standard laboratory diet and water were provided *ad libitum*.

### 2.3. Animals and Treatment

**Figure 1:**
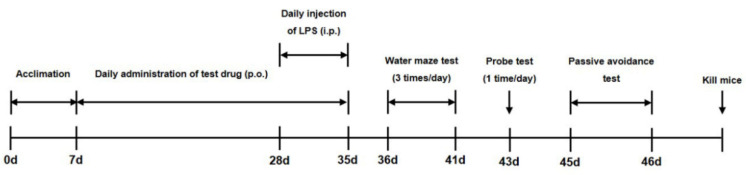
Overall experimental timeline for assessment of the effect of banhasasim-tang (BHS) on the cognitive function of mice with lipopolysaccharide-induced neuroinflammation. ICR mice were acclimated to the facility for 7 days (0–7 days), and the test drug BHS was administered by mouth (p.o.) for 4 weeks (7–35 days). During the final week, lipopolysaccharide (LPS; experimental groups) or PBS (control groups) was given intraperitoneally (i.p.). Between days 36 and 46, neurocognitive testing was held to collect endpoints. Mice were euthanized after completion of all testing.

Mice were acclimatized to the facility for 7 days before experimental procedures were initiated. Animals were randomly divided into five groups with eight mice in each group: Normal (phosphate-buffered saline (PBS) + vehicle), LPS (negative control; LPS + vehicle), BHS 150 (LPS + BHS 150 mg/kg each day), BHS 300 (LPS + BHS 300 mg/kg each day), and Indo (positive control; LPS + Indo 10 mg/kg each day). Mice were injected intraperitoneally (i.p.) with LPS (400 μg/kg) or the same volume of PBS once daily for 7 days after the drug treatment. BHS 150, BHS 300, and Indo were administered once per day for 28 days, whereas the Normal and LPS groups received vehicle only (Figure 1).

### 2.4. Morris Water Maze and Probe Test

After 4 weeks of drug administration, the Morris water maze (MWM) test was performed to investigate the memory and learning abilities of five groups mice. The water maze consisted of a 1.2-m-diameter rubber pool filled with opaque water (reverse osmosis water diluted with black paint) at room temperature containing a 10-cm-diameter hidden platform. An overhead video camera connected to the SMART video tracking software (Panlab, Barcelona, Spain) was used to track and record the swimming trajectory of all groups of mice. Each trial was performed for 1 min until the mouse climbed onto the hidden platform target, and the escape latency and escape distance of the mouse were recorded. Two days after the navigation test, the probe test was conducted without the platform in the northeast quadrant of the rubber pool. Each mouse was placed into the pool to swim for the same period of time. The percentage of time spent in the northeast quadrant and the number of times each mouse crossed the platform area were analyzed. The above two behavioral tests were conducted in reference to previous research [14].

### 2.5. Passive Avoidance Test

The passive avoidance (PA) test is widely used to test memory. The test was performed using a Shuttle Box Avoidance Basic Test Package (Med Associates Inc., Fairfax, VT, USA) composed of four reaction chambers. The apparatus was divided into two rooms, an illuminated room and a dark room, that were connected together via an automatic door. The dark room was equipped with a grid floor through which an electronic shock could be delivered. In the training trial, the mice were initially placed into the light room; after 30 s of habituation, the automatic door was opened to allow the mice to move to the dark room. The door was closed quickly after the mice passed into the dark room, and an electric shock (0.35 mA, 3 s) was delivered. The test trial was performed the next day, during which each mouse was reevaluated in the same manner without an electronic shock. The latency times for the mice moving from the light room to the dark room were recorded up to a maximum of 3 min. The PA test was conducted in accordance with a previous study [15].

### 2.6. Nissl Staining

Mice were euthanized, and brains were collected and postfixed in 10% formalin solution for 24 h. Tissue samples of five groups mice were washed with PBS for 10 min. Paraffin-embedded brain sections were placed in a rotary microtome, and 5-μm-thick consecutive coronal slices were prepared until the hippocampal surface was reached. Brain section slices were dewaxed in xylene twice for 20 min and rehydrated in a series of ethanol grades (100%, 95%, 90%, 80%, 70% and 50%) for 1 min each. Then, sample slices were stained with a warm 0.5% cresyl violet solution for 10 min, rinsed with distilled water, and dehydrated in a series of ethanol grades (50%, 70%, 80%, 90%, 95%, and 100%). Tissue slices were cleared in xylene twice for 20 min each time, and slides were mounted with glass cover slips [16]. Images were captured using a microscope. Normal neurons were quantified at 200–400× magnification. Three fields were randomly selected in each slice. The number of normal neurons with clear Nissl granules stained by cresyl violet and distinct nuclei were counted, and the average number was calculated for analysis.

### 2.7. Immunohistochemical Staining

Brains were paraffin-embedded and cut into 5-μm sections. Brain tissue section slices were immersed in xylene twice for 5 min each at room temperature and rehydrated in 100%, 95%, 90%, 80%, 70%, and 50% ethanol for 1 min each. For antigen epitopes, section slices were boiled in 10 mM sodium citrate buffer and incubated in 3% H_2_O_2_ for 30 min to block endogenous peroxidase, followed by incubation with 5% normal goat serum for 1 h at room temperature. After blocking, the sections were incubated overnight at 4 °C with primary antibodies against activated microglia using ionized calcium-binding adaptor molecule 1 (Iba-1) and activated astrocytes via glial fibrillary acidic protein (GFAP), and then they were washed in PBS with 0.1% tween 20 (PBS-T) and incubated with peroxidase-conjugated secondary antibody for 1 h at room temperature. Sections were washed in PBS-T and detected using a REAL EnVision detection system (WAKO) for 5 min before being rinsed in PBS-T to stop detection. Sections were stained with hematoxylin to label nuclei and then observed under a microscope. Microglial and astrocyte activity was characterized by an increase in the number of cells and an alteration in cell morphology that led to an increase in the level of Iba-1 or GFAP labeling [17].

### 2.8. Western Blotting for Protein Expression of the Mouse Brain and BV2 Cells

To prepare the samples for Western blotting, mouse brain tissues or BV2 cells were lysed in radioimmunoprecipitation assay (RIPA) buffer (Millipore) and incubated on ice for 1 h. After centrifugation at 15,000× *g*, supernatant was acquired and normalized using Bradford’s reagent, followed by denaturation at 95 °C. Equivalent protein samples (20 μg) were applied to 8%–12% sodium dodecyl sulfate–polyacrylamide gel electrophoresis and transferred to PVDF membranes using transfer buffer. Membranes were incubated with 3% BSA for 1 h at room temperature for blocking of nonspecific binding sites and incubated overnight with various specific primary antibodies at 4 °C. The membranes were then washed four times using tris-buffered saline with 0.1% tween 20 (TBS-T), incubated with horseradish peroxidase (HRP)-conjugated secondary antibodies for 1 h at room temperature, and then detected on Chemidoc using ECL solution. The information about the various primary and secondary antibodies is listed in Table 2. Each protein band was normalized by β-actin and quantified using ImageJ software.

### 2.9. RNA Extraction and RT-qPCR

Total RNA was isolated using the easy-BLUE RNA extraction kit according to the manufacturer’s protocol. A total of 1 μg RNA was reverse-transcribed into cDNA using AccuPower RT PreMix; qPCR oligonucleotide primers for mouse brain and microglial cell cDNA are indicated in Table 3 [18]. qPCR reactions were conducted in triplicate in 20 μL volumes with the following reagents: 0.3 μM final concentration of each primer (1 μL each for forward and reverse), 10 μL of AccuPower 2× Greenstar qPCR master mix, 5 μL template DNA, and 3 μL RNase-free water. The PCR cycle was as follows: 95 °C for 10 min; 40 cycles of 95 °C for 20 s; and 60 °C (tumor necrosis factor (TNF)-α, interleukin (IL)-6, inducible nitric oxide synthase (iNOS), cyclooxygenase (COX)-2), 55 °C (IL-1β), or 61 °C (heme oxygenase (HO)-1) for 40 s. At the end of each experiment, a melting curve analysis was performed to confirm that a single product per primer pair was amplified [18]. Amplification and analysis were performed using QuantStudio 6 Flex Real-Time PCR System (Thermo Scientific, Rockford, IL, USA), and each sample was compared using the relative C_T_ method. Fold changes in gene expression were determined relative to the blank control after normalization to β-actin expression using the 2^−ΔΔCt^ method.

### 2.10. Cell Culture, Stimulation, and BHS Treatment

The mouse microglia BV2 cell line was obtained from Prof. Kyoungho Suk at Kyungpook National University (Daegu, Korea). BV2 cells were maintained in complete DMEM (containing 1% antibiotics and 10% FBS). Cells were incubated in humidified 5% CO_2_ atmosphere at 37 °C [19]. All experiments were conducted the day after cell seeding in 6-, 24-, or 96-well culture plates. To stimulate an inflammatory response, the culture medium was replaced with fresh DMEM (containing 1% antibiotics), and 100 ng/mL of LPS [19] was added in the presence or absence of BHS (1–500 μg/mL).

### 2.11. Cell Viability Assay, Griess Assay, and ELISA for the Inflammatory Cytokine Secretion

To determine cell viability, nitrite production, and cytokine secretion, cells were plated in 24- or 96-well culture plates (5 × 10^5^ cells/mL) and were incubated with various concentrations of BHS and 100 ng/mL LPS for 24 h (for Griess assay and ELISA) or 48 h (for viability assay). To test cell viability, the CCK solution was added and incubated for an additional 1 h. Cell viability was calculated as optical density using an ELISA plate reader (450 nm; SpectraMax i3; Molecular Devices, San Jose, CA, USA). To analyze nitric oxide (NO) production, 100 μL of Griess reagent was added to each well. After incubation for 5 min, the absorbance at 570 nm was measured in the plate reader. For ELISA, culture medium was obtained, and cell debris was removed by centrifugation. Experiments were performed using ELISA kits according to the manufacturer’s standard protocol. Secretion of each inflammatory cytokine was evaluated by the color intensity after treatment with the substrate 3,3′,5,5′-tetramethylbenzidine (TMB) solution at an absorbance read at 450 nm on the plate reader.

### 2.12. Preparation of Cytoplasmic and Nuclear Protein Extracts

Cytoplasmic and nuclear extracts of the BV2 cells were separated according to the manufacturer’s standard protocol with NE-PER Nuclear and Cytoplasmic Extraction Reagents (Thermo Scientific). The extracts were stored in a deep freezer at −80 °C, and the subsequent procedure was the same as that used for Western blotting of whole cell lysate.

### 2.13. Statistical Analysis

Results are expressed as mean ± standard error of the mean (SEM). Statistical significance was determined by one-way analysis of variance (ANOVA) followed by Dunnett’s test after comparing LPS and each treated group. Statistical significance was defined as # *p* < 0.05 (vs. Normal or Control), * *p* < 0.05, ** *p* < 0.001, and † *p* < 0.0001 (vs. LPS).

## 3. Results

### 3.1. Mitigation Effects of BHS Treatment on LPS-Induced Cognitive Impairment in Mice

**Figure 2:**
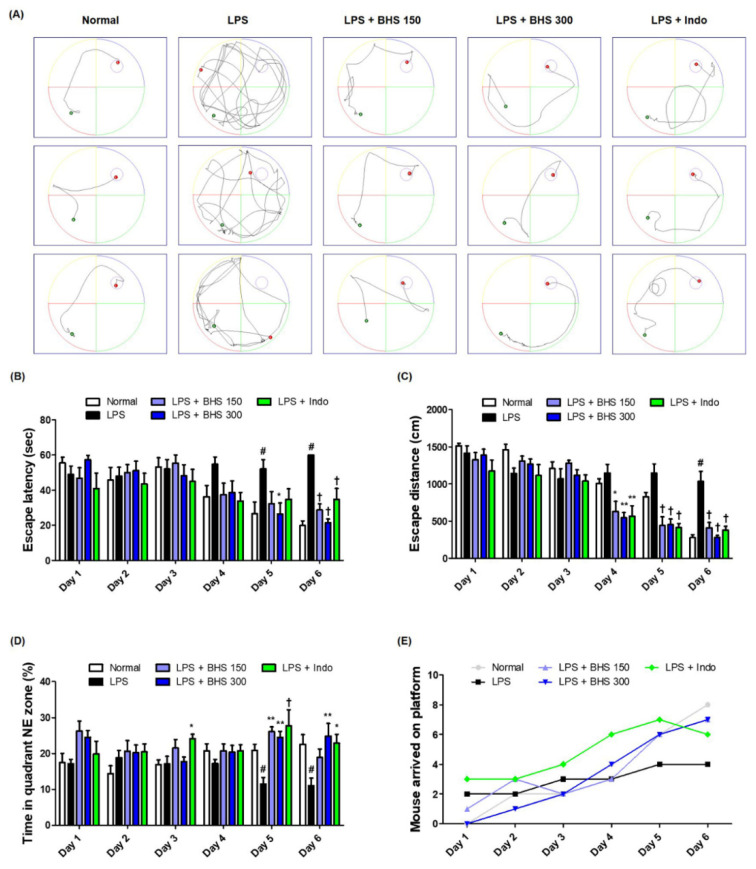
Effects of BHS on LPS-induced cognitive impairment. Memory function of the mice was determined by (**A**) swimming trajectory, (**B**) escape latency, (**C**) escape distance, (**D**) time spent in NE quadrant, and (**E**) number of mice arriving at platform in the MWM test. Data represent the mean ± SEM from *n* = 8 mice. # *p* < 0.05 (vs. Normal), * *p* < 0.05, ** *p* < 0.001, and † *p* < 0.0001 (vs. LPS) by one-way ANOVA followed by Dunnett’s test. BHS, banhasasim-tang; Indo, indomethacin; LPS, lipopolysaccharide; MWM, Morris water maze; NE, northeast.

To determine the influence of BHS on LPS-induced memory loss, we performed an MWM test and measured the escape latency time and distance from the water maze in order to examine the ability of mice to learn their location and exhibit spatial memory recall. Mice in the Normal group showed relatively fast and good learning, while the LPS-injected mice showed poor learning compared with other groups. LPS-injected mice having received BHS arrived at the platform with a simpler trajectory than those of the LPS group without BHS when exiting the water maze (Figure 2A). During the six-day training period, the average escape latency (Figure 2B) and the distance the mice swam to escape to the platform (Figure 2C) greatly decreased. The administration of BHS significantly reduced both the escape latency time and distance at the end of the training period in a manner that implicated dependence on the capacity between low and high doses. In addition, the anti-inflammatory drug Indo, used as a positive control, also showed an inhibitory effect on LPS-induced memory loss. As shown in Figure 2D,E, analysis of the duration that the mice stayed in the northeast quadrant where the platform was located and how many mice successfully escaped the water maze within 60 s suggested that BHS had an excellent effect on conservation of memory in this mouse model.

### 3.2. Inhibitory Effects of BHS Treatment on Memory Loss Induced by LPS in Probe Test and PA Test

**Figure 3:**
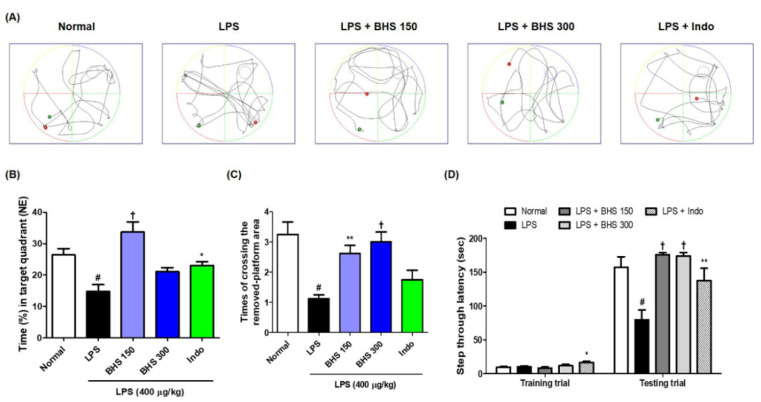
Effects of BHS on mouse memory loss induced by LPS injection, as assessed by probe test (**A**–**C**) and PA test (**D**). (**A**) Swimming trajectory, (**B**) time spent in NE quadrant, and (**C**) times the mice crossed the removed-platform area in the probe test. (**D**) Step-through latency of training and test trial in PA test. Data represent the mean ± SEM from *n* = 8 mice. # *p* < 0.05 (vs. Normal), * *p* < 0.05, ** *p* < 0.001, and † *p* < 0.0001 (vs. LPS) by ANOVA with Dunnett’s post-hoc analysis. BHS, banhasasim-tang (at 150 or 300 mg/kg per day); Indo, indomethacin; LPS, lipopolysaccharide; MWM, Morris water maze; NE, northeast; PA, passive avoidance test.

After the last day of the MWM test, we conducted a probe test to analyze the time spent in the target quadrant area and the number of times the mice passed the previous platform location. BHS-treated mice spent a higher percentage of time in the northeast quadrants than mice in the LPS groups without BHS (Figure 3A,B), and the number of times that the mice passed by the platform’s previous location was significantly higher in the BHS-treated LPS groups than in the untreated LPS groups (Figure 3A,C).

The PA test was conducted to assess how long a mouse was able to remember its location. The BHS-treated group with LPS displayed an increased step-through latency compared with that of the untreated LPS group (Figure 3D). In addition, the Indo group also showed significant memory improvement in both tests.

### 3.3. BHS Treatment Prevents LPS-Induced Neuronal Damage in the Mouse Brain

**Figure 4:**
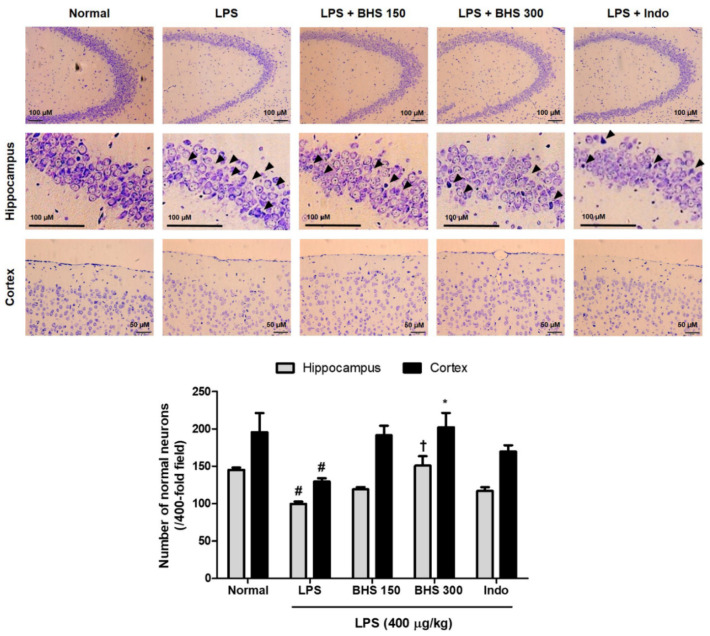
Effects of BHS on LPS-induced neuronal damage and histopathology in mice. Nissl staining images and the number of normal neurons in the hippocampus and cortex of each group of mice. Scale bars = 100 μm for hippocampus and 50 μm for cortex. Data represent mean ± SEM. Significant differences compared with the Normal group are indicated by # *p* < 0.05. Significant differences compared with the LPS group are indicated by * *p* < 0.05 and † *p* < 0.0001. BHS, banhasasim-tang (at 150 or 300 mg/kg per day); Indo, indomethacin; LPS, lipopolysaccharide.

To investigate the effect of the BHS decoction on mouse neurons, we analyzed histopathologic changes using Nissl staining with cresyl violet. Loss of Nissl substances in neurons due to LPS injection was apparent compared with saline-treated normal mice rich in Nissl corpuscles. Administration of BHS to the LPS group significantly increased Nissl substances in brain cells and increased the number of neurons with normal morphology in the hippocampus and cortex (Figure 4). It was also observed that Indo had a neuroprotective effect on LPS-treated mice.

### 3.4. Inhibitory Effects of BHS Administration on Neuroinflammation via LPS-Induced Activation of Iba-1 and GFAP

**Figure 5:**
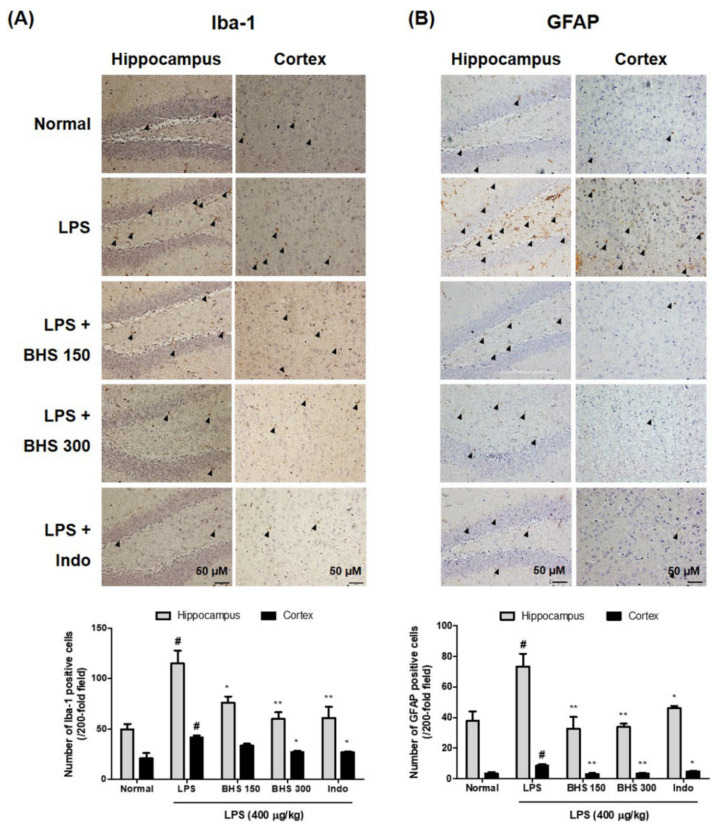
Effects of BHS on LPS-induced neuroinflammation in the mouse brain. Immunostaining of (**A**) Iba-1 or (**B**) GFAP proteins in the hippocampus and cortex was performed in 5-μm sections of mouse brains with primary antibodies. Arrowheads indicate positive staining. Scale bars = 50 μm. Data represent means ± SEM. # *p* < 0.05 compared with the Normal group; * *p* < 0.05 and ** *p* < 0.001 compared with the LPS group. BHS, banhasasim-tang (at 150 or 300 mg/kg per day); GFAP, glial fibrillary acidic protein; Iba-1, ionized calcium-binding adaptor molecule 1; Indo, indomethacin; LPS, lipopolysaccharide.

We detected the expression of Iba-1 and GFAP, markers of microglia and astrocyte activation in the brain, through immunohistochemistry, which showed activation of microglia and astrocytes as well as occurrence of neuroinflammation in mice injected with LPS. As shown in the 3,3′-diaminobenzidine (DAB)-stained images of Figure 5A,B Iba-1-reactive and GFAP-reactive cell numbers were very high in LPS-injected mice compared with normal mice. BHS administration in LPS-injected mice displayed decreased activation of microglia and astrocytes. Specifically, the positive cell numbers of the dentate gyrus, cornu ammonis (CA)1 and CA3 parts of the hippocampus, and the cortex were significantly decreased in LPS-injected mice treated with BHS compared with those without BHS treatment.

### 3.5. BHS Treatment Inhibits Protein Production Related to Amyloidogenesis and Neuroinflammation, Activation of NF-κB/MAPK, and Expression of Inflammatory Mediator mRNAs

**Figure 6:**
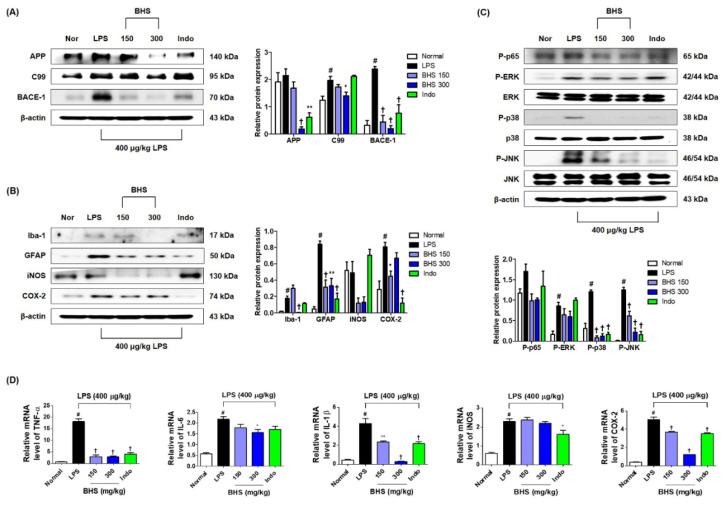
Effects of BHS on LPS-induced amyloidogenesis, neuroinflammation, NF-κB/MAPK activation, and mRNA expression of inflammatory mediators in the mouse brain. Protein expression levels of (**A**) APP, C99, and BACE-1 and (**B**) Iba-1, GFAP, iNOS, and COX-2 were detected by Western blotting. (**C**) Activation of NF-κB and MAPK pathways. (**D**) mRNA expression of inflammatory mediators was analyzed by RT-qPCR. Histograms show protein or mRNA levels relative to those of an internal control such as β-actin or total-type protein. Data represent means ± SEM. # *p* < 0.05 compared with the Normal group; * *p* < 0.05, ** *p* < 0.001, and † *p* < 0.0001 compared with the LPS group. APP, amyloid-β precursor protein; BACE-1, β-secretase-1; BHS, banhasasim-tang; C99, β-CTF fragment of APP; COX-2, cyclooxygenase-2; ERK, extracellular signal-regulated kinase; GFAP, glial fibrillary acidic protein; HO-1, heme oxygenase; Iba-1, ionized calcium-binding adaptor molecule 1; Indo, indomethacin; iNOS, inducible nitric oxide synthase; IL-1β, interleukin 1β; IL-6, interleukin 6; JNK, c-Jun NH2-terminal kinase; LPS, lipopolysaccharide; MAPK, mitogen-activated protein kinase; NF-κB, nuclear factor κB; TNF-α, tumor necrosis factor alpha.

To investigate whether BHS influenced amyloidogenesis inhibition in the mouse brain, we performed Western blotting using mouse hippocampal tissue. As shown in Figure 6A, BHS administration inhibited the elevation of amyloid-β precursor protein (APP), β-CTF fragment of APP (C99), and β-secretase-1 (BACE-1) expression levels by LPS injection. We also examined the inhibitory effect of BHS on neuroinflammation in the brains of LPS-injected mice. Treatment with LPS elevated the number of activated microglia (Iba-1 staining) and activated astrocytes (GFAP staining) as well as the expression of inflammatory proteins, including iNOS and COX-2, but these levels were effectively suppressed by administration of BHS (Figure 6B). However, the expression of COX-2 did not appear to be dose-dependent.

Activation of nuclear factor (NF)-κB and mitogen-activated protein kinase (MAPK) have been implicated in amyloidogenesis and neuroinflammation [20,21,22]. Thus, we examined the activation levels of NF-κB and MAPK by measuring phosphorylation of p65, extracellular signal-regulated kinase (ERK), p38, and c-Jun NH_2_-terminal kinase (JNK). Phosphorylation of p65 was decreased by treatment with 150 and 300 mg/kg BHS (Figure 6C). Phosphorylation of ERK, p38, and JNK was also significantly inhibited by BHS administration in a dose-dependent manner (Figure 6C). Administration of BHS additionally inhibited LPS-induced upregulation of mRNA expression of inflammatory cytokines, including TNF-α, IL-6, and IL-1β, and of oxidative stress inflammatory enzymes, including iNOS and COX-2 (Figure 6D).

### 3.6. Effects of BHS on the Cell Viability of BV2 Microglia, Secretion of Nitric Oxide, and Production of Inflammatory Cytokines

**Figure 7:**
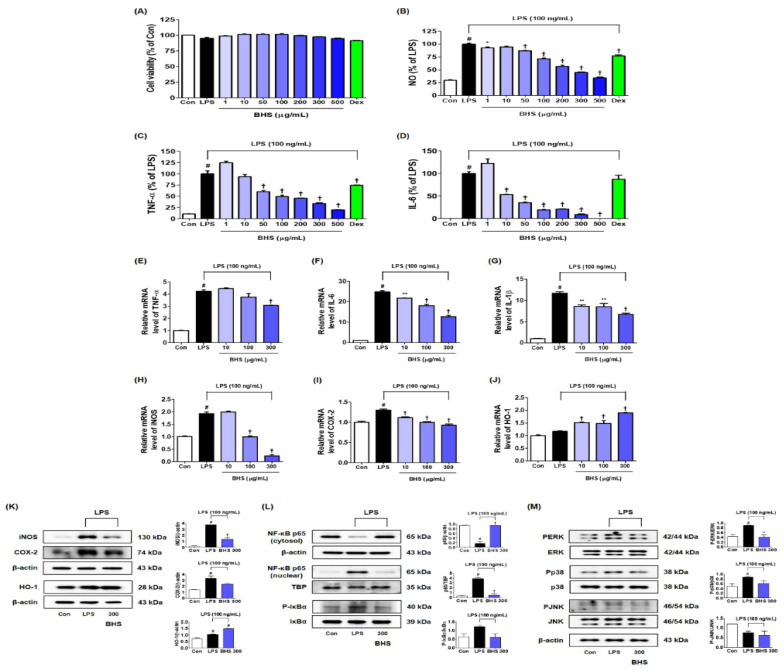
Effects of BHS on the inflammatory response of LPS-stimulated BV2 microglial cells: (**A**) cell viability; (**B**) secretion of NO; (**C**,**D**) inflammatory cytokine protein expression; (**E**–**G**) inflammatory cytokine mRNA expression; (**H**–**J**) mRNA expression and (**K**) protein production of iNOS, COX-2, and HO-1; and regulatory effects of BHS on the activation of (**L**) NF-κB and (**M**) MAPK pathways. Control cells were incubated with vehicle alone. The histograms show protein and mRNA levels relative to those of β-actin, TATA-box-binding protein, or total-type protein. Data represent means ± SEM of determinations from three independent experiments. Statistical significance is defined as # *p* < 0.05 (vs. Control) and * *p* < 0.05, ** *p* < 0.001, and † *p* < 0.0001 (vs. LPS). BHS, banhasasim-tang; COX-2, cyclooxygenase-2; Dex, dexamethasone; ERK, extracellular signal-regulated kinase; GFAP, glial fibrillary acidic protein; HO-1, heme oxygenase; Iba-1, ionized calcium-binding adaptor molecule 1; IL-1β, interleukin 1β; IL-6, interleukin 6; JNK, c-Jun NH2-terminal kinase; LPS, lipopolysaccharide; MAPK, mitogen-activated protein kinase; NF-κB, nuclear factor κB; NO, nitric oxide; TNF-α, tumor necrosis factor alpha.

Cell viability tests using the CCK solution showed no cytotoxicity to BV2 microglia cells when treated with 1–500 μg/mL of BHS and 10 μg/mL of Dex (Figure 7A). Griess assays were carried out to investigate the effects of BHS on nitric oxide (NO) secretion. In this and subsequent experiments, the efficacy of BHS was compared with that of 10 μM Dex, which was used as a positive control during in vitro tests. BV2 cells showed a sharp increase in NO secretion after LPS stimulation and showed a pattern of inhibition upon pretreatment with BHS or Dex (Figure 7B). To determine the efficacy of BHS for the regulation of neuroinflammatory reaction at the cellular level, the secretion of proinflammatory cytokines was investigated. LPS-induced cytokine secretion of both TNF-α and IL-6 was decreased after pretreatment with various concentrations of BHS (Figure 7C,D). Likewise, TNF-α, IL-6, and IL-1β mRNA expression was significantly inhibited by BHS treatment in a concentration-dependent manner (Figure 7E–G).

### 3.7. Effects of BHS Pretreatment on the Expression of iNOS and COX-2 Enzymes and Anti-inflammatory Factor HO-1

At the mRNA level, the expression of iNOS and COX-2 was increased by LPS, and pretreatment with BHS inhibited this in a concentration-dependent manner (Figure 7H,I). As shown in Figure 7J, the mRNA level of the anti-inflammatory factor HO-1 was significantly increased after treatment with 10 μg/mL or more BHS (vs. Control). BHS pretreatment similarly significantly inhibited protein expression of iNOS and COX-2 and increased the induction of HO-1 protein (Figure 7K).

### 3.8. Inhibitory Effects of BHS on the Activation of Inflammatory Pathways NF-κB and MAPK

We investigated the effects of BHS on the activation of NF-κB and MAPK, which are typical pathways of inflammation. Western blotting showed that pretreatment with 300 μg/mL BHS significantly inhibited translocation of p65 from the cytoplasm into the nucleus as well as degradation and phosphorylation of inhibitor of NF-κB alpha (IκBα), indicative of the activation of the NF-κB pathway (Figure 7L). Furthermore, BHS treatment effectively inhibited phosphorylation of ERK and p38 but not JNK in LPS-stimulated BV2 cells (Figure 7M).

## 4. Discussion

Recent studies have demonstrated that systemic inflammation by LPS injection causes brain injury in mice, resulting in a decline in spatial memory that, for instance, greatly increases escape latency to find a hidden platform in behavioral tests [23,24]. Such unregulated inflammatory responses and brain injury have been reported as important risk factors of neurodegenerative diseases such as Alzheimer’s, Parkinson’s, and Huntington’s diseases. BHS is an important herbal medication that has long been used in East Asia; although several of its beneficial pharmacological functions have been reported in recent studies, its effects on systemic inflammatory reactions or cognitive impairment have not been studied. Thus, we investigated the effects of BHS on the learning and memory of LPS-injected mice. Our water maze and PA results show that BHS significantly reduces memory loss imposed by LPS injection and positively affects learning ability and spatial memory (Figure 2 and Figure 3). Mice given the anti-inflammatory drug Indo also showed an overall improvement in memory function, demonstrating that inhibition of systemic inflammation affects memory ability in this model.

Several studies have indicated that the hippocampus and cortex play an important role in learning and memory and demonstrated that the structure and function of pyramidal cells in the hippocampus and cortex are correlated with learning and memory functions [25,26]. Histopathological observation of the mouse brain through Nissl staining in the current study revealed damage to pyramidal cells in the hippocampus and cortex after LPS administration, characterized by widespread loss of the Nissl corpuscle and normal neurons. Our Nissl staining results therefore show that this pathological change in the brains of the mice given BHS is inhibited in a large part and indicates that the number of normal neurons of the hippocampus and cortex increases significantly and dose-dependently (Figure 4).

Some studies have recently shown that systemic injection of LPS causes neuroinflammatory reactions with direct damage to the blood–brain barrier, causing amyloidogenesis and memory deficiency [27]. Moreover, LPS activates neuronal and glial cells, which secrete neurotoxic factors and cause inflammation in the brain. Repeated administration of LPS causes inflammation and amyloidogenesis due to increased Aβ formation and can lead to cognitive degradation, spatial memory failure, and learning impairment during the process of Alzheimer’s disease [28,29]. Therefore, we analyzed the expression of Iba-1 and GFAP, activation markers of microglia and astrocytes, respectively, through immunohistochemistry and found that BHS treatment significantly inhibits overactivation by LPS injection (Figure 5).

NF-κB is activated by inflammatory intermediates and oxidative stress, and transcription of genes for expression of APP and BACE-1 is affected by NF-κB [21]. MAPK is also an important inflammatory mechanism associated with neuroinflammation and apoptosis by various stimuli [20]. In particular, phosphorylation of p38 MAPK is involved in cognitive impairment related to neuroinflammation [22]. Accordingly, we investigated the effects of BHS on the expression of amyloidogenesis, neuroinflammation, and the NF-κB and MAPK pathways. We found that administration of BHS inhibits the LPS-induced expression of amyloidogenesis-related proteins and also reduces the generation levels of neuroinflammatory markers in mouse hippocampal tissues (Figure 6A,B). This also indicates that BHS treatment is capable of controlling the activation of NF-κB and MAPK pathways by inhibiting the phosphorylation of p65, ERK, p38, and JNK (Figure 6C). Moreover, BHS treatment effectively inhibits inflammatory cytokine and enzyme mRNA expression by LPS in the mouse hippocampus (Figure 6D).

Microglia, brain-specific macrophages, are resident in the brain and form the frontline defense of the innate immune system. However, excessive activation of microglia leads to neuronal death, brain injury, and release of a variety of neuroinflammation mediators like NO, iNOS, COX-2, and inflammatory cytokines. The reduction of these inflammatory factors via modulation of microglia activation is a method of significant importance in the treatment of neuroinflammation [30]. In this study, we observed the efficacy of BHS in mice, as well as the anti-neuroinflammatory effects of BHS and its molecular mechanisms in LPS-stimulated BV2 microglia. NO is a free radical implicated in the neuroinflammatory reaction process in the CNS [31]. It is synthesized from L-arginine by iNOS, and its generation and the induction of HO-1 have a close negative correlation [32]. Here, we found that BHS pretreatment effectively inhibited the secretion of NO and expression of iNOS in BV2 cells stimulated by LPS and significantly increased the induction of HO-1 at both protein and mRNA levels (Figure 7B,H,J,K). NF-κB and MAPK signaling pathways have been associated with the pathogenesis of various diseases in CNS. NF-κB transcription factor is a key regulator in inflammatory responses [33], and MAPK has a critical role in the production of inflammatory mediators [34]. Therefore, we investigated whether the inhibitory effects of BHS on proinflammatory factors is achieved through the NF-κB and MAPK pathways. Our results showed that BHS treatment inhibited activation of NF-κB via suppression of IκBα degradation and phosphorylation, and it repressed phosphorylation of MAPK (Figure 7L,M).

In this study we demonstrated the efficacy of BHS on cognitive impairment caused by neuroinflammation, and we explored its inhibitory mechanism. However, in addition to neuroinflammation, vascular disorder is also an important risk factor for cognitive decline or dementia [35,36]; multidimensional prevention strategies for these risk factors are an effective way to slow down the progression of neurodegenerative diseases. Therefore, it is desirable that future research on the treatment of neurodegenerative diseases will be conducted as multidimensional efficacy studies on various risk factors, and we will also explore the efficacy of BHS in various ways and prove its value as a candidate applicable to clinical research.

## 5. Conclusions

In summary, this study demonstrated the mitigating effect of BHS on LPS-induced cognitive impairment of mice. BHS improved learning and spatial memory function and protected against neuronal damage in the mouse brain through inhibition of neuroinflammation. In addition, BHS attenuated the histopathological changes in the mouse brain and inhibited the expression of activation markers of microglia and astrocytes. BHS treatment also inhibited overproduction of neuroinflammatory mediators and blocked LPS-induced activation of inflammatory pathways in mouse brain tissue and BV2 microglial cells. Based on these results, BHS decoction has strong potential as a therapeutic candidate for neurodegenerative diseases.

## Figures and Tables

**Table 1:** The herbal ingredients and composition ratio of banhasasim-tang (BHS).

Scientific Name of Herbs	Medicinal Parts	Composition Ratio (%)
*Pinellia ternata* (Thunb.) Makino	Tuber	24.5
*Scutellaria baicalensis* Georgi	Root	14.6
*Panax ginseng* C.A. Mey.	Root	14.6
*Glycyrrhiza uralensis* Fisch.	Root and rhizome	14.6
*Zingiber officinale* Roscoe	Rhizome	12.2
*Coptis japonica* (Thunb.) Makino	Rhizome	4.9
*Ziziphus jujuba* Mill.	Fruit	14.6

**Table 2:** Primary and secondary antibodies use for Western blot analysis.

Antibody	Corporation	Product No.	RRID	Dilution Rate
APP	Cell Signaling	#2450	AB_490857	1:1000
C99	Millipore	#MABN380	AB_2714163	1:1000
BACE-1	Cell Signaling	#5606	AB_1903900	1:1000
β-actin	Santa Cruz	#SC-47778	AB_626632	1:1000
Iba-1	Novus Biologicals	#NB100-1028	AB_521594	1:1000
GFAP	Santa Cruz	#SC-33673	AB_627673	1:1000
iNOS	Cell Signaling	#13120	AB_2687529	1:1000
COX-2	Cell Signaling	#4842	AB_2085144	1:1000
P-NF-κB p65	Cell Signaling	#3033	AB_331284	1:1000
P-ERK	Cell Signaling	#4377	AB_331775	1:1000
ERK	Cell Signaling	#9102	AB_330744	1:1000
P-p38	Cell Signaling	#9211	AB_331641	1:1000
p38	Cell Signaling	#9212	AB_330713	1:1000
P-JNK	Cell Signaling	#9251	AB_331659	1:1000
JNK	Cell Signaling	#9252	AB_2250373	1:1000
HO-1	Cell Signaling	#82206	AB_2799989	1:1000
NF-κB p65	Cell Signaling	#8242	AB_10859369	1:1000
TBP	Cell Signaling	#8515	AB_10949159	1:1000
P-IκBα	Cell Signaling	#2859	AB_561111	1:1000
IκBα	Cell Signaling	#4814	AB_390781	1:1000
2nd anti-mouse	Cell Signaling	#7076	AB_330924	1:5000
2nd anti-rabbit	Cell Signaling	#7074	AB_2099233	1:5000
2nd anti-goat	Santa Cruz	#SC-2020	AB_631728	1:5000

**Table 3:** Primers used for RT-qPCR.

Gene	Primer Sequence	PCR Product (bp)
TNF-α	F: 5′-TTCTGTCTACTGAACTTCGGGGTGATCGGTCC-3′	354
	R: 5′-GTATGAGATAGCAAATCGGCTGACGGTGTGGG-3′	
IL-6	F: 5′-TCCAGTTGCCTTCTTGGGAC-3′	140
	R: 5′-GTGTAATTAAGCCTCCGACTTG-3′	
IL-1β	F: 5′-ATGGCAACTGTTCCTGAACTCAACT-3′	563
	R: 5′-CAGGACAGGTATAGATTCTTTCCTTT-3′	
iNOS	F: 5′-GGCAGCCTGTGAGACCTTTG-3′	72
	R: 5′-GCATTGGAAGTGAAGCGTTTC-3′	
COX-2	F: 5′-TGAGTACCGCAAACGCTTCTC-3′	151
	R: 5′-TGGACGAGGTTTTTCCACCAG-3′	
HO-1	F: 5′-TGAAGGAGGCCACCAAGGAGG-3′	373
	R: 5′-AGAGGTCACCCAGGTAGCGGG-3′	
β-actin	F: 5′-AGAGGGAAATCGTGCGTGAC-3′	138
	R: 5′-CAATAGTGATGACCTGGCCGT-3′	

F, forward; R, reverse; TNF-α, tumor necrosis factor alpha; IL-6, interleukin 6; IL-1β, interleukin 1 beta; iNOS, inducible nitric oxide synthase; COX, cyclooxygenase; HO, heme oxygenase.
